# Concentrations of digestible and metabolizable energy are greater in pistachio blanks than in wheat middlings, but some amino acids in pistachio blanks have reduced digestibility compared with soybean meal when fed to growing pigs

**DOI:** 10.1093/jas/skaf454

**Published:** 2025-12-30

**Authors:** Yeonwoo Kim, Su A Lee, Hans H Stein

**Affiliations:** Division of Nutritional Sciences, University of Illinois at Urbana-Champaign, Urbana 61801, IL; Department of Animal Sciences, University of Illinois at Urbana-Champaign, Urbana 61801, IL; Division of Nutritional Sciences, University of Illinois at Urbana-Champaign, Urbana 61801, IL

**Keywords:** amino acids, energy, growing pigs, pistachio blanks, wheat middlings

## Abstract

Pistachio blanks are a mixture of pistachio byproducts that consist of undersized or unripe seeds and hard shells that may potentially be used as a high-fiber feed ingredient in diets for pigs, but there is limited information about the nutritional value of pistachio blanks when fed to pigs. Therefore, two experiments were conducted to determine the chemical composition and energy and amino acid (**AA**) digestibility of pistachio blanks fed to growing pigs. In experiment 1, the objective was to test the null hypothesis that apparent total tract digestibility (**ATTD**) of gross energy and digestible energy (**DE**), and metabolizable energy (**ME**) in pistachio blanks are not different from values obtained in wheat middlings when fed to growing pigs. Twenty-four growing pigs with an initial body weight of 23.01 ± 0.90 kg were individually housed in metabolism crates. A basal diet contained corn and soybean meal as the sole sources of energy, and two diets contained corn, SBM, and 30% pistachio blanks, or corn, SBM, and 30% wheat middlings. Twenty-four pigs were allotted to the three diets with eight replicate pigs per diet. Feces and urine samples were quantitatively collected for 4 d after seven days of adaptation. Results indicated that the ATTD of dry matter and gross energy and DE and ME were greater (*P *< 0.05) in pistachio blanks than in wheat middlings. In experiment 2, nine growing barrows (initial body weight: 22.67 ± 0.93 kg) were equipped with a T-cannula in the distal ileum and allotted to a triplicated 3 × 3 Latin square design with three diets and three 7-d periods, for nine replicate pigs per treatment. Two diets contained pistachio blanks or soybean meal as the sole source of AA and a nitrogen-free diet was also formulated. The apparent ileal digestibility of dry matter, crude protein, indispensable AA, and dispensable AA was greater (*P *< 0.05) in soybean meal than in pistachio blanks. The standardized ileal digestibility (**SID**) of Ile, Leu, Phe, Val, Ala, Asp, Gly, Pro, and Ser was not different between pistachio blanks and soybean meal, but the SID of Arg, His, Lys, and Met was greater (*P *< 0.05) in soybean meal than in the pistachio blanks. In contrast, the SID of Thr and Trp was greater in pistachio blanks than in in soybean meal. In conclusion, pistachio blanks contain more DE and ME than wheat middlings, but the SID of some, but not all, indispensable AA is less than in soybean meal.

## Teaser

Pistachio blanks are a high-fiber co-product from the pistachio industry and may be included in diets for growing pigs as a high-fiber ingredient.

## Introduction

Pistachio production has expanded in recent years and future expansion is expected. California is the leading producer of pistachio in the United States (Tootelian, 2023), and production in CA is expected to increase by up to 50% over the next 5 yr. Pistachio blanks are co-products of pistachio processing and are discarded due to their hollow shells or immature nuts, which make them unusable as human food. These immature or defective nuts are separated from other nuts by flotation, because blanks typically float in water, and separated pistachio shells and other materials that also float in water, are collected together with the immature nuts. Utilizing co-products such as pistachio blanks aligns with sustainable livestock production goals by reducing feed costs and minimizing waste. A similar co-product, pistachio shell powder, was recently demonstrated to provide significant amounts of energy in diets for sows (Kim et al., 2024). However, limited data exist for pistachio blanks and the digestibility of amino acids (**AA**) and energy in pistachio blanks fed to pigs has not been reported. Therefore, two experiments were conducted to test the hypothesis that apparent total tract digestibility (**ATTD**) of gross energy (**GE**), and the concentration of digestible energy (**DE**) and metabolizable energy (**ME**) is greater in pistachio blanks than in wheat middlings. The second hypothesis was that the apparent ileal digestibility (**AID**) and standardized ileal digestibility (**SID**) of AA in pistachio blanks are not different from AID and SID of AA in soybean meal (**SBM**).

## Materials and Methods

The Institutional Animal Care and Use Committee at the University of Illinois reviewed and approved the protocol for two experiments. Pigs in both experiments were the offspring of Line 800 boars and Camborough females (Pig Improvement Company, Hendersonville, TN, USA).

### Experimental diets

The same batch of pistachio blanks was used in both experiments. This batch was procured from Wonderful LLC (Los Angeles, CA, USA; Table 1), and the blanks were dried and ground to approximately 475 microns prior to being delivered to the University of Illinois. Conventional dehulled SBM was procured from Solae LLC (Gibson City, IL, USA), whereas wheat middlings were procured from ADM Milling (Mendota, IL, USA). Locally grown corn was obtained from the University of Illinois Feed Mill (Urbana, IL, USA), and this batch was used in all diets for experiment 1 (Table 1).

**Table 1.** Analyzed nutrient composition of feed ingredients, as-is basis

Item	Corn	Soybean meal	Pistachio blanks	Wheat middlings
**Dry matter, %**	85.71	88.34	93.92	88.24
**Ash, %**	1.05	5.99	2.92	5.29
**Crude protein, %**	8.02	46.72	6.11	16.76
**Acid hydrolyzed ether extract, %**	3.28	2.57	4.35	3.44
**Gross energy, kcal/kg**	3,792	4,132	4,538	4,101
**Total dietary fiber, %**	13.30	20.50	81.10	42.70
** Soluble dietary fiber**	2.20	3.10	6.50	1.40
** Insoluble dietary fiber**	11.10	17.40	74.60	41.30
** Glucose, %**	0.28	<0.05	0.62	0.28
** Sucrose, %**	1.09	7.16	0.61	2.03
** Maltose, %**	<0.05	0.26	<0.05	1.07
** Fructose, %**	0.20	0.11	0.44	0.20
** Stachyose, %**	<0.05	5.40	<0.05	<0.05
** Raffinose, %**	0.12	1.04	0.37	1.25
** Starch, %**	61.00	–	–	19.61
**Indispensable amino acids, %**				
** Arginine**	0.34	3.22	1.30	1.28
** Histidine**	0.23	1.24	0.12	0.50
** Isoleucine**	0.29	2.26	0.20	0.60
** Leucine**	0.92	3.57	0.32	1.08
** Lysine**	0.26	2.82	0.32	0.79
** Methionine**	0.15	0.61	0.07	0.26
** Phenylalanine**	0.37	2.36	0.22	0.71
** Threonine**	0.26	1.73	0.17	0.57
** Tryptophan**	0.04	0.50	0.03	0.13
** Valine**	0.37	2.34	0.28	0.87
**Dispensable amino acids, %**				
** Alanine**	0.56	1.98	0.26	0.87
** Aspartic acid**	0.50	5.15	0.69	1.33
** Cysteine**	0.16	0.60	0.09	0.38
** Glutamic acid**	1.43	8.45	0.85	3.19
** Glycine**	0.29	1.91	0.22	0.98
** Proline**	0.68	2.28	0.36	1.04
** Serine**	0.32	1.88	0.22	0.63
** Tyrosine**	0.20	1.63	0.13	0.45
**Total amino acids, %**	7.37	44.53	5.85	15.66

In experiment 1, three diets were formulated (Table 2). The basal diet was based on corn and SBM and two additional diets were formulated by mixing 30% pistachio blanks or 30% wheat middling with corn and SBM, while maintaining the same ratio between corn and SBM as in the basal diet. Vitamins and minerals were included in all diets to meet or exceed the estimated requirements for growing pigs (NRC, 2012). In experiment 2, two diets that contained pistachio blanks or SBM as the sole source of AA were formulated, and a nitrogen-free diet was used to determine basal endogenous losses of crude protein (**CP**) and AA (Table 3). Vitamins and minerals were included in all diets to meet or exceed current requirement estimates (NRC, 2012). All diets also contained 0.4% chromic oxide as an indigestible marker.

**Table 2.** Composition (as-is basis) of experimental diets, experiment 1

Item	Corn	Pistachio blanks	Wheat middlings
**Ingredient, %**			
** Ground corn**	61.40	42.38	42.67
** Soybean meal**	36.10	24.92	25.08
** Pistachio blanks**	–	30.00	–
** Wheat middling**	–	–	30.00
** Dicalcium phosphate**	0.90	1.25	0.15
** Ground limestone**	0.70	0.55	1.20
** Sodium chloride**	0.40	0.40	0.40
**Vitamin micromineral premix^1^**	0.50	0.50	0.50
**Analyzed composition^2^**			
** Dry matter, %**	88.32	89.63	87.91
** Crude protein, %**	21.80	16.20	20.15
** Ash, %**	3.55	4.52	4.24
** Gross energy, kcal/kg**	3,885	4,023	3,929

1 The vitamin-micromineral premix provided the following quantities of vitamins and micro minerals per kg of complete diet: vitamin A as retinyl acetate, 10,622 IU; vitamin D_3_ as cholecalciferol, 1,660 IU; vitamin E as selenium yeast, 66 IU; vitamin K as menadione nicotinamide bisulfate, 1.40 mg; thiamin as thiamine mononitrate, 1.08 mg; riboflavin, 6.49 mg; pyridoxine as pyridoxine hydrochloride, 0.98 mg; vitamin B_12_, 0.03 mg; _D-_pantothenic acid as _D-_calcium pantothenate, 23.2 mg; niacin, 43.4 mg; folic acid, 1.56 mg; biotin, 0.44 mg; Cu, 20 mg as copper chloride; Fe, 123 mg as iron sulfate; I, 1.24 mg as ethylenediamine dihydriodide; Mn, 59.4 mg as manganese hydroxychloride; Se, 0.27 mg as sodium selenite and selenium yeast; and Zn, 124.7 mg as zinc hydroxychloride.

2 All diets were formulated to contain 0.63% Ca and 0.31% standardized total tract digestible P (NRC, 2012).

**Table 3.** Composition (as-is basis) of experimental diets, experiment 2

Ingredient, %	Pistachio blanks	Soybean meal	N-free
** Pistachio blanks**	40.00	–	–
** Soybean meal**	–	40.00	–
** Soybean oil**	3.00	3.00	4.00
** Solka floc**	–	–	4.00
** Dicalcium phosphate**	1.20	1.20	2.15
** Limestone**	0.68	0.68	0.45
** Lactose**	20.00	20.00	20.00
** Chromic oxide**	0.40	0.40	0.40
** Cornstarch**	33.82	33.82	67.55
** Magnesium oxide**	–	–	0.10
** Potassium carbonate**	–	–	0.40
** Sodium chloride**	0.40	0.40	0.40
** Vitamin-micromineral premix^1^**	0.50	0.50	0.50
**Analyzed nutrient composition**			
** Dry matter, %**	92.20	90.38	90.24
** Gross energy, kcal/kg**	4,038	3,975	3,651
** Crude protein, %**	5.48	20.19	–
** Ash, %**	3.23	5.15	2.86
**Indispensable amino acids, %**			
** Arginine**	0.37	1.51	0.01
** Histidine**	0.04	0.57	0.01
** Isoleucine**	0.06	1.04	0.01
** Leucine**	0.09	1.67	0.02
** Lysine**	0.10	1.34	0.02
** Methionine**	0.02	0.29	0.00
** Phenylalanine**	0.06	1.12	0.01
** Threonine**	0.05	0.81	0.01
** Tryptophan**	0.02	0.25	0.02
** Valine**	0.07	1.08	0.01
** Total**	0.88	9.68	0.12
**Dispensable amino acids, %**			
** Alanine**	0.08	0.93	0.01
** Aspartic acid**	0.21	2.41	0.02
** Cysteine**	0.03	0.29	0.01
** Glutamic acid**	0.23	4.02	0.03
** Glycine**	0.06	0.90	0.01
** Proline**	0.12	1.05	0.01
** Serine**	0.06	0.91	0.01
** Tyrosine**	0.04	0.75	0.01
** Total**	0.83	11.26	0.11
**Total amino acids, %**	1.71	20.94	0.23

1 The vitamin-micromineral premix provided the following quantities of vitamins and micro minerals per kg of complete diet: vitamin A as retinyl acetate, 10,622 IU; vitamin D_3_ as cholecalciferol, 1,660 IU; vitamin E as selenium yeast, 66 IU; vitamin K as menadione nicotinamide bisulfate, 1.40 mg; thiamin as thiamine mononitrate, 1.08 mg; riboflavin, 6.49 mg; pyridoxine as pyridoxine hydrochloride, 0.98 mg; vitamin B_12_, 0.03 mg; _D-_pantothenic acid as _D-_calcium pantothenate, 23.2 mg; niacin, 43.4 mg; folic acid, 1.56 mg; biotin, 0.44 mg; Cu, 20 mg as copper chloride; Fe, 123 mg as iron sulfate; I, 1.24 mg as ethylenediamine dihydriodide; Mn, 59.4 mg as manganese hydroxychloride; Se, 0.27 mg as sodium selenite and selenium yeast; and Zn, 124.7 mg as zinc hydroxychloride.

### Animals and housing

In experiment 1, 24 growing barrows (initial body weight: 23.01 ± 0.90 kg) were allotted to a completely randomized design with three diets and eight replicate pigs per diet. Pigs were housed individually in metabolism crates (0.81 m × 1.52 m) that were equipped with a self-feeder, a nipple waterer, and a slatted floor. A screen and a urine pan were placed under the slatted floor to allow for the total, but separate, collection of urine and fecal materials. Pigs were limit fed at 3.0 times the maintenance requirement for ME (i.e., 197 kcal/kg × weight^0.60^; NRC, 2012), which is slightly below expected voluntary energy intake to make sure pigs could finish their daily allotments of feed. Equal daily meals were provided at 0800 and 1600 h. Throughout the experiment, pigs had free access to water. Pigs were fed experimental diets for 13 d, with feed consumption recorded daily.

In experiment 2, nine growing barrows (initial body weight: 22.67 ± 0.93 kg) were equipped with a T-cannula in the distal ileum (Stein et al., 1998). Pigs were allotted to a triplicated 3 × 3 Latin square design with three diets and three 7-d periods (Kim and Stein, 2009). Therefore, there were nine replicate pigs per treatment. Pigs were housed in individual pens (1.2 × 1.5 m) in an environmentally controlled room with the ambient temperature maintained between 20 and 24°C. Pens had smooth sides and fully slatted tribar floors. A feeder and a nipple drinker were installed in each pen. Water was available at all times and feed was provided at a daily level of 3.4 times the estimated requirement for ME, because this level was assumed to be close to the ad libitum intake. Because of the use of the indigestible marker in the diets used in this experiment, it was not critical that pigs consumed every meal completely.

### Sample collection

In experiment 1, the initial seven days were considered the adaptation period to the diet. Urine and fecal materials were collected from the feed provided during the following four days according to standard procedures using the marker-to-marker approach (Adeola, 2001). Therefore, collection of feces started when the first color marker (i.e., the start marker), which was included in the morning meal on day 8, appeared in the feces, and fecal collections ceased when the stop marker, which was provided in the morning meal on day 12, appeared. Because experimental diets were fed for 13 d, all pigs had time to pass the stop-marker before the end of the experiment. Most pigs passed the stop-marker within 24 h of feeding (i.e., in the morning of day 13), and the remaining pigs all passed the stop-marker within 36 h of feeding (i.e., in the evening of day 13). Fecal samples and 10% of the collected urine were stored at −20°C immediately after collection.

In experiment 2, each period of the Latin square lasted 7 d, and the initial 5 d of each period were considered the adaptation period to the diet, whereas ileal digesta were collected on days 6 and 7 for 8 h each day (Stein et al., 1998). By attaching a plastic bag to the opened cannula barrel using a cable tie, digesta that flowed into the bag were collected. Bags were replaced every time they were filled with digesta or at least once every 30 min. Digesta samples were stored at −20°C immediately after collection to prevent bacterial degradation of the AA in the digesta (Lee et al., 2021). Pig weights were recorded at the beginning of each period and at the conclusion of the experiment. The amount of feed supplied each day was recorded. On the completion of one experimental period, animals were deprived of feed overnight and the following morning, a new experimental diet was offered.

### Chemical analysis

In both experiments, all diet and ingredient samples were analyzed for dry matter (**DM**; method 927.05; AOAC Int., 2019) and ash (method 942.05; AOAC Int., 2019). Gross energy was analyzed using an isoperibol bomb calorimeter (Model 6400, Parr Instruments, Moline, IL, USA). Benzoic acid was used for calibration. The concentration of nitrogen was analyzed by combustion (method 990.03; AOAC Int., 2019) using a LECO FP628 analyzer (LECO Corp., Saint Joseph, MI, USA) with subsequent calculation of CP as nitrogen × 6.25. Ingredients and diets from experiment 2 were analyzed for AA (method 982.30 E (a, b, c); AOAC Int., 2019) and total starch was analyzed in corn and wheat middlings using the glucoamylase procedure (method 979.10; AOAC Int., 2019). Ingredient samples were also analyzed for acid hydrolyzed ether extract using the acid hydrolysis filter bag technique (Ankom HCl Hydrolysis System; Ankom Technology, Macedon, NY, USA) followed by crude fat extraction using petroleum ether (method 2003.06, AOAC Int., 2019; Ankom XT15 Extractor; Ankom Technology, Macedon, NY, USA). Glucose, fructose, maltose, sucrose, stachyose, and raffinose were analyzed in ingredients using high-performance liquid chromatography (Dionex App Notes 21 and 92). Insoluble dietary fiber and soluble dietary fiber were analyzed in ingredients according to method 991.43 (AOAC Int., 2019) using the Ankom Dietary Fiber Analyzer (Ankom Technology, Macedon, NY, USA). Total dietary fiber was calculated as the sum of insoluble dietary fiber and soluble dietary fiber.

At the conclusion of experiment 1, urine samples were thawed and mixed within animal and diet, and a sub-sample was lyophilized before analysis (Kim et al., 2009). Fecal samples were thawed and mixed within pig and diet and dried in a 55°C forced air-drying oven (HMH750, Thermo Scientific; Waltham, MA, USA) for seven days prior to analysis. Fecal samples were ground through a 1-mm screen using a hammermill (model: MM4; Schutte Buffalo, NY, USA), mixed, and sub-sampled for analysis. Fecal samples were analyzed for DM and GE as explained for diet analysis. Urine samples were analyzed for GE (Kim et al., 2009).

In experiment 2, ileal digesta samples were thawed and lyophilized (Gamma 1-16 LSCplus, IMA Life, Tonawanda, NY, USA; Lagos and Stein, 2019) and then ground. Samples were analyzed for DM, CP, and AA as described for diets and ingredients. The concentration of chromium was determined in diets and ileal digesta using the Inductive Coupled Plasma Atomic Emission Spectrometric method (method 990.08; AOAC Int., 2019).

### Calculations

In experiment 1, the ATTD of GE and DM were calculated for each diet, and the DE and ME in each diet were calculated as well (NRC, 2012). The DE and ME in the corn-soybean meal mixture were divided by the inclusion rate of corn and soybean meal in that diet to calculate the DE and ME in the mixture. The contribution of DE and ME from corn and soybean meal to the two test diets was calculated and the DE and ME in the pistachio blanks and wheat middlings were calculated by difference (Adeola, 2001). The ATTD of GE in pistachio blanks and wheat middlings was also calculated by difference.

In experiment 2, the AID and SID were calculated for CP and AA in the two diets containing pistachio blanks or SBM (Stein et al., 2007). The basal endogenous losses of CP and AA were calculated from pigs fed the nitrogen-free diet (Stein et al., 2007). The values calculated for diets also represented the values for each ingredient, because the two test ingredients were the only AA-containing ingredients in the diets.

### Statistical analysis

Data were analyzed using the MIXED procedure of SAS (SAS Institute Inc., Cary, NC). Homogeneity of the variances and normality among treatments were confirmed using UNIVARIATE procedure, and this procedure was also used to identify outliers using Internally Studentized Residuals (Tukey, 1977). Mean values were calculated using the LSMeans statement and if the model was significant, means were separated using the PDIFF statement with Tukey’s adjustment. Pig was the experimental unit, and results were considered significant at *P* ≤ 0.05 and 0.05 ≤ *P* < 0.10 was considered a tendency.

## Results

For both experiments, all pigs consumed their diets throughout the experiment without apparent problems. The analyzed concentrations of GE and nutrients in diets were in accordance with values calculated from the ingredients.

### Experiment 1

Feed intake by pigs fed the three experimental diets was not different, but GE intake tended (*P *< 0.10) to be greater for pigs fed the pistachio diet than pigs fed the basal diet (Table 4). Dry feces output and GE fecal output from pigs fed the pistachio blanks diet were less (*P *< 0.05) than from pigs fed the wheat middlings diet, but not different from that of pigs fed the basal diet. Urine output and GE urine output were not different among diets. The ATTD of DM and GE were less (*P *< 0.05) for the wheat middlings diet than for the other diets. The DE and ME were also less (*P *< 0.05) in the wheat middlings diet compared with the other diets, but no differences were observed between the basal diet and the pistachio blank diet. Likewise, when calculating the values for only the ingredients, ATTD of GE and DE and ME were less (*P *< 0.05) in wheat middlings than in pistachio blanks. The DE: GE and the ME: GE were also less (*P *< 0.05) in wheat middlings than in pistachio blanks, whereas ME: DE tended (*P *< 0.10) to be less in wheat middlings than in pistachio blanks.

**Table 4.** Apparent total tract digestibility (ATTD) of dry matter (DM) and gross energy (GE) and concentrations of digestible energy (DE) and metabolizable energy (ME) in experimental diets fed to growing pigs, experiment 1^1^, as-fed basis

Item	Basal diet	Pistachio blank	Wheat middlings	SEM	*P*-value
**Intake**					
** Diet, kg/day**	1.26	1.28	1.27	0.02	0.733
** GE, Mcal/day**	0.49	0.52	0.50	0.09	0.096
**Fecal excretion**					
** Dry feces output, kg/day**	0.14^b^	0.18^b^	0.24^a^	0.01	<0.001
** GE, kcal/d**	630^b^	801^b^	1,072^a^	63	<0.001
**Urine excretion**					
** Urine output, kg/day**	6.87	7.17	4.98	1.01	0.276
** GE, kcal/d**	231	229	228	22	0.994
**ATTD of DM, %**	88.47^a^	85.72^a^	79.61^b^	1.11	<0.001
**ATTD of GE, %**	87.16^a^	84.61^a^	78.42^b^	1.15	<0.001
**Energy in diets**					
** DE, kcal/kg**	3,387^a^	3,403^a^	3,081^b^	46	<0.001
** DE in DM, kcal/kg**	3,835^a^	3,797^a^	3,505^b^	52	<0.001
** ME, kcal/kg**	3,201^a^	3,225^a^	2,901^b^	55	0.001
** ME in DM, kcal/kg**	3,624^a^	3,598^a^	3,300^b^	61	0.002
**Energy in ingredients**					
** ATTD of GE**	–	78.29	59.17	3.64	0.003
** DE, kcal/kg**	–	3,553	2,427	164	0.001
** DE in DM, kcal/kg**	–	3,783	2,750	175	0.002
** ME, kcal/kg**	–	3,385	2,255	171	0.001
** ME in DM, kcal/kg**	–	3,604	2,556	183	0.002
** DE: GE, %**	–	78.29	59.17	3.64	0.003
** ME: DE, %**	–	95.04	92.89	0.79	0.074
** ME: GE, %**	–	74.59	54.99	3.80	0.003

1 Each least squares mean represents eight observations per diet.

a,b Within a row, means without a common superscript differ (*P *< 0.05).

### Experiment 2

The AID of DM, CP, all indispensable AA, and all dispensable AA was greater (*P *< 0.01) in SBM than in pistachio blanks (Table 5). However, the SID of Ile, Leu, Phe, Val, Ala, Asp, Gly, Pro, and Ser was not different between pistachio blanks and SBM (Table 6), but, the SID of Arg, His, Lys, and Met was greater (*P *< 0.05) in SBM than in pistachio blanks. The SID of crude protein, Glu, and Tyr also tended (*P* < 0.10) to be greater in soybean meal than in pistachio blanks, whereas the SID of Thr and Trp was greater (*P* < 0.05) in pistahcio blanks than in soybean meal.

**Table 5.** Apparent ileal digestibility (%) of dry matter, crude protein and amino acids (AA) in pistachio blanks and soybean meal, experiment 2^1^

Item	Pistachio blanks	Soybean meal	SEM	*P*-value
**Dry matter**	62.2	72.8	1.95	0.003
**Crude protein**	3.1	84.6	4.05	<0.001
**Indispensable amino acids**				
** Arginine**	74.7	92.2	0.75	<0.001
** Histidine**	−13.4	87.2	7.19	<0.001
** Isoleucine**	−6.1	86.6	8.58	<0.001
** Leucine**	−19.7	86.2	11.57	<0.001
** Lysine**	−23.8	85.9	8.38	<0.001
** Methionine**	3.7	88.4	5.54	<0.001
** Phenylalanine**	−13.3	86.4	11.12	<0.001
** Threonine**	−58.4	80.1	10.56	<0.001
** Tryptophan**	24.8	85.6	5.13	<0.001
** Valine**	−21.8	84.9	10.83	<0.001
**Dispensable amino acids**				
** Alanine**	−32.1	81.5	10.50	<0.001
** Aspartic acid**	31.6	83.4	4.62	<0.001
** Cysteine**	−32.5	72.4	11.64	<0.001
** Glutamic acid**	19.4	87.1	5.92	<0.001
** Glycine**	−135.0	73.6	8.72	<0.001
** Proline**	−153.1	76.7	34.32	<0.001
** Serine**	−21.3	85.2	6.24	<0.001
** Tyrosine**	−19.7	88.0	6.75	<0.001
**Total**	−1.8	84.8	4.58	<0.001

1 Each least squares mean represents nine observations for soybeans and 8 observations for pistachio blanks.

**Table 6.** Standardized ileal digestibility (%) of crude protein and amino acids (AA) in pistachio blanks and soybean meal, experiment 2^1^^,2^

Item	Pistachio blanks	Soybean meal	SEM	*P*-value
**Crude protein**	82.7	91.2	3.09	0.86
**Indispensable amino acids**				
** Arginine**	88.7	95.9	0.80	<0.001
** Histidine**	61.6	92.0	5.03	<0.001
** Isoleucine**	97.5	91.9	4.63	0.406
** Leucine**	101.5	91.9	5.09	0.219
** Lysine**	56.3	91.1	5.28	<0.001
** Methionine**	73.0	92.7	4.19	0.009
** Phenylalanine**	99.6	91.7	4.49	0.241
** Threonine**	127.7	90.8	6.92	0.005
** Tryptophan**	107.5	91.7	3.96	0.020
** Valine**	98.8	91.8	5.29	0.374
**Dispensable amino acids**				
** Alanine**	93.4	91.1	5.64	0.771
** Aspartic acid**	86.3	87.7	2.92	0.733
** Cysteine**	103.6	85.1	6.89	0.058
** Glutamic acid**	81.8	90.2	3.09	0.071
** Glycine**	101.6	88.9	9.30	0.358
** Proline**	22.6	97.7	35.58	0.150
** Serine**	92.0	92.2	4.68	0.977
** Tyrosine**	84.2	93.0	3.55	0.097
**Total**	84.2	91.4	3.42	0.178

1 Each least squares mean represents nine observations for soybean meal and eight observations for pistachio blanks.

2 Standardized ileal digestibility was calculated by correcting the apparent ileal digestibility for basal ileal endogenous losses. Basal ileal endogenous losses were determined (g/kg of dry matter intake) as crude protein, 15.57; Arg, 0.56; His, 0.30; Ile, 0.62; Leu, 1.06; Lys, 0.76; Met, 0.14; Phe, 0.65; Thr, 0.96; Trp, 0.17; Val, 0.82; Ala, 0.98; Asp, 1.16; Cys, 0.41; Glu, 1.39; Gly, 1.53; Pro, 2.44; Ser, 0.71; and Tyr, 0.42.

## Discussion

The GE in all diets used in the experiment was in agreement with calculated values, which indicated that errors in diet mixing, subsampling, and GE analysis likely were minimal. High-fiber co-products from the human food industry are often used in diets fed to livestock (Zijlstra and Beltranena, 2013; Stein et al., 2015). According to NRC (2012), wheat co-products may be classified as wheat bran, wheat shorts, or wheat middlings, and in other parts of the world, the term “wheat pollard” is also used (Fanelli et al., 2024). The concentration of proximate nutrients in wheat middlings appears to be almost identical to that in wheat bran (NRC, 2012), and the naming of wheat co-products is often more influenced by tradition and local customs than by differences in nutrient composition (Fanelli et al., 2024). The wheat coproduct used in this experiment was purchased as “wheat middlings” and the nutrient composition of the ingredient was within the range of values for wheat middlings from the U.S. used in previous experiments (Casas et al., 2018; Espinosa et al., 2024). However, due to the similarity in composition between wheat bran and wheat middlings, it is understood that the ingredient might also have been classified as “wheat bran.”

Pistachio shell powder is a co-product from the pistachio industry, and gestating sows were able to ferment the fiber in pistachio shell powder and obtain energy from the ingredient (Kim et al., 2024). There are, however, no data for the nutritional value of pistachio blanks, and the present experiments were conducted to fill this gap. Pistachio shell powder mostly consists of insoluble dietary fiber, whereas pistachio blanks contain more CP and lipids, but less total dietary fiber, than pistachio shell powder. This difference may be due to the presence of immature nuts in pistachio blanks, which contain some CP and lipids.

In experiment 1, the determined DE and ME in the basal diet were in very good agreement with expected values (NRC, 2012), which gives confidence that values for pistachio blanks and wheat middlings are also accurate. The ATTD of GE and DM for wheat middlings obtained in this experiment concur with values reported by Casas et al. (2018), but DE and ME were less than some reported values (NRC, 2012). However, these values also concur with values reported by Casas et al. (2018) and differences in DE and ME among different sources of wheat middlings have been reported (Huang et al., 2014; Espinosa et al., 2024). The observation that pistachio blanks contain almost 3,400 kcal/kg of ME indicates that despite the high fiber concentration, pigs are able to efficiently convert the energy in fiber into ME. Thus, results of this experiment are consistent with results with pistachio shell powder fed to sows (Kim et al., 2024). It is remarkable that a high-fiber ingredient such as pistachio blanks provides more energy than wheat middlings, suggesting that the insoluble fiber in pistachio shell powder and pistachio blanks is more fermentable than the fiber in wheat middlings.

For pistachio shell powder, it was demonstrated that the major fiber component is xylan polysaccharides, but unlike wheat middlings where the major fiber component is arabinoxylan (Jaworski et al., 2015), there appears to be limited arabinoxylan in pistachio fiber (Kim et al., 2024). It is, therefore, likely that other fiber components, such as xyloglucans may be the major source in pistachio fiber, which may explain its greater fermentability compared with fiber in wheat middlings. It is possible that the fiber in pistachio shell powder and pistachio blanks may positively affect the microbiome in the hindgut of pigs, which in turn allows the microbes to ferment the fiber. Future research should, therefore, be directed at determining possible impacts of pistachio fiber on hind gut microbiome.

It is apparent that pistachio blanks are low in AA, which resulted in the AID of most AA to be very low. Some negative AID values occurred because pigs had greater endogenous losses of these AA than the quantities absorbed. However, because SID values are calculated by correcting AID values for basal endogenous losses, there is no impact of endogenous AA losses on SID values, making SID a better measure of the AA digestibility of a feed ingredient (Stein et al., 2007). The SID of AA in SBM was in very good agreement with expected values (NRC, 2012). The observation that the SID of some AA in pistachio blanks was less than in SBM indicates that growing pigs are less efficient in digesting the protein in pistachio blanks than in SBM. However, it is well established that high levels of dietary fiber have negative impacts on ileal AA digestibility (Mosenthin et al., 1994; Souffrant, 2001; Zhang et al., 2024) and it is, therefore, likely that the high concentration of fiber in pistachio blanks contributed to the reduced SID of AA. The low SID of Lys in pistachio blanks is likely due to heat damage during drying and grinding of the ingredient (Almeida et al., 2013).

The reason the SID of AA in pistachio blanks was compared with the SID of AA in SBM is that SBM is a common source of AA in diets for pigs and the SID of AA in SBM is very well characterized, which makes SBM an appropriate ingredient for comparison. However, it is recognized that it may also be relevant to compare the SID of AA in pistachio blanks with the SID of AA in another high-fiber ingredient such as wheat middlings or soybean hulls.

One of the consequences of determining SID of AA in an ingredient with a low concentration of AA is that the endogenous losses of AA may have a greater influence on the AID of AA in the low-AA ingredient compared with an ingredient with a greater concentration of AA (Stein et al., 2005). However, because SID values were calculated, the impact of basal endogenous losses on digestibility of AA was eliminated, and values that are representative of the ingredients were calculated (Stein et al., 2005). In addition, calculating SID of AA in low-AA ingredients using the direct procedure, as was done in this experiment, provides values that are not different from values calculated using the difference procedure where it will be possible to maintain a constant protein level among diets (Oliveira et al., 2020).

Nevertheless, the low concentration of AA in pistachio blanks and the low SID of some AA indicate that whereas pistachio blanks appear to be an excellent source of energy and fiber in diets for growing pigs, the ingredient has low AA value. Therefore, diets containing pistachio blanks will need to be supplemented with additional sources of AA to meet the requirements for all AA in pigs.

## Conclusion

Results of these experiments indicate that the fiber in pistachio blanks is highly fermentable by growing pigs, which results in high values for DE and ME. However, the low concentration of digestible AA indicates that pistachio blanks primarily provide energy to the diets, and a supplemental source of AA must be added to provide sufficient AA to the diets.

## Abbreviations:

AA: amino acids
AID: apparent ileal digestibility
ATTD: apparent total tract digestibility
CP: crude protein
DE: digestible energy
DM: dry matter
GE: gross energy
ME: metabolizable energy
SBM: soybean meal
SID: standard ileal digestibility
